# Analysis of linear accelerator-based fractionated stereotactic radiotherapy in brain metastases: efficacy, safety, and dose tolerances

**DOI:** 10.3389/fonc.2024.1471004

**Published:** 2024-11-21

**Authors:** Yuhong Li, Huiying Ma, Rui Hua, Tingting Wang, Naixin Ding, Liping Deng, Xiaomin Lu, Wei Chen

**Affiliations:** ^1^ Department of Radiation Oncology, Affiliated Cancer Hospital of Nanjing Medical University, Jiangsu Cancer Hospital, Jiangsu Institute of Cancer Research, Nanjing, China; ^2^ Department of Radiation Oncology, The First People's Hospital of Jiande, Hangzhou, China; ^3^ Department of Oncology, Nanjing Drum Tower Hospital, Clinical College of Nanjing Drum Tower Hospital, Nanjing Medical University, Nanjing, China; ^4^ Department of Oncology, Affiliated Haian Hospital of Nantong University, Nantong, China

**Keywords:** brain metastases, linear accelerators, fractionated stereotactic radiotherapy, dose-effect relation, biologically effective dose, targeted therapy

## Abstract

**Objective:**

To assess the efficacy and safety of linear accelerator-based fractionated stereotactic radiotherapy (LINAC-FSRT) in patients with brain metastases (BM).

**Methods:**

We retrospectively analyzed 214 patients treated with LINAC-FSRT, categorized based on biologically effective dose (BED10, *α*/*β* = 10) into two groups (≤55 Gy, >55 Gy). Stratified analyses were conducted based on targeted therapy to compare survival outcomes. To examine brain tissue dose-tolerance volume, patients were divided into two groups: the standard Hypofractionated Treatment Effects in the Clinic (HyTEC) protocol group and an adjusted HyTEC protocol group where dose-volume restrictions exclude the planning target volume (PTV).

**Results:**

Results as of December 2023 showed median intracranial progression-free survival (iPFS) at 12.4 months, with median overall survival (OS) not reached and a one-year local control (LC) rate of 68.7%. Mild to moderate toxicity affected 17.3% of patients, while severe toxicity occurred in 2.8%. Multivariate Cox analysis indicated that uncontrolled extracranial disease significantly reduced iPFS (HR = 2.692, 95%CI:1.880–3.853, *P* < 0.001) and OS (HR = 3.063, 95%CI:1.987–4.722, *P* < 0.001). BED10 >55 Gy (HR = 0.656, 95%CI:0.431–0.998, *P* = 0.049) improved OS, showing statistical significance (*P* = 0.037) without affecting iPFS or CNS toxicity (*P* = 0.127, *P* = 0.091). Stratified analysis highlighted nearly significant OS improvements with high-dose FSRT and targeted therapy (*P* = 0.054), while concurrent therapy markedly enhanced iPFS (*P* = 0.027). No significant differences were observed in intracranial local failure (ILF—which represents progression in previously treated areas during follow-up), one-year LC rates, iPFS, or OS between dose-volume groups. Adjusting HyTEC volume restrictions did not significantly increase CNS adverse reactions (*P* = 0.889).

**Conclusions:**

LINAC-FSRT is safe and effective in BM. BED10>55 Gy notably enhances OS post-LINAC-FSRT and may benefit LC. High BED10 FSRT with targeted therapy likely boosts synergy, and concurrent targeted therapy significantly improves iPFS. Diminishing dose volume constraints at different fractions based on the HyTEC guidelines is feasible.

## 1 Introduction

Brain metastases (BM) are one of the most prevalent intracranial cancers in adults. Autopsy investigations show that up to 40% of patients with malignant tumors acquire BM. Typically, these patients have poor long-term survival prospects, with only 2.4% surviving more than five years (1). Due to probable neurocognitive deficits, whole brain radiation (WBRT) is being replaced with stereotactic radiosurgery (SRS) and fractional stereotactic radiotherapy (FSRT) (2, 3). Stereotactic radiation (SRT) precisely delivers high doses while sparing surrounding healthy brain tissue with steep dose gradients at target margins. Current recommendations prescribe SRS for patients with 1-4 BM and a satisfactory baseline condition (2). However, for larger tumors (diameter > 2 cm), the side effects of SRS may be severe. In contrast, FSRT employs various fractions to maximize radiobiological effects, efficiently regulating BM while limiting the risk of radiation necrosis (RN) (4, 5). Technologically, the widespread usage of linear accelerators (LINAC) in recent years has maintained the therapeutic efficacy of FSRT, providing higher operational convenience and cost-effectiveness than classic gamma knife SRT (6, 7). As a result, FSRT has become a more realistic treatment option for large-volume BM (8). In the present time, the integration of tyrosine kinase inhibitors (TKIs) targeted therapy with FSRT is broadening its therapeutic uses (9–13). Targeted therapy also includes cyclin-dependent kinase inhibitors, which have been preliminarily shown to be safe and effective in combination with stereotactic brain radiotherapy as well (14).

Multiple studies demonstrate that FSRT has a reduced occurrence of RN following treatment compared to SRS; yet, complications from fractionated therapies remain problematic (4, 5). Research has persistently investigated the correlation between the quantities of brain tissue exposed to radiation during SRT and neurotoxic side effects, revealing substantial associations between volumes receiving 12 Gy and 24 Gy and the incidence of RN (4, 15). The Hypofractionated Treatment Effects in the Clinic (HyTEC) recommendations have delineated specific brain tissue tolerance volumes for one, three, and five fractions of FSRT, aimed at minimizing toxic effects (16, 17). Nonetheless, the majority of research have examined a restricted array of FSRT fractionation schemes (mostly 2-5 fractions), and the dose-volume metrics assessed differ significantly. As FSRT therapy techniques evolve, there is an imperative to expand research to enhance clinical standards.

This retrospective study examined data from brain metastasis patients treated with LINAC-FSRT at our institution, assessing its safety and efficacy. It specifically utilized biologically effective dose (BED) to evaluate dose-response relationships across LINAC-FSRT fractionation schemes (2-12 fractions), investigated synergies with systemic targeted therapies, and adhered to HyTEC principles to establish more suitable dose-volume tolerance standards for our LINAC-FSRT, thereby improving dosing precision for BM treatment.

## 2 Materials and methods

### 2.1 Patient selection and follow-up

Approval for ethical considerations was granted by the Institutional Ethics Committee of Jiangsu Cancer Hospital (Approval No: KY-2024-078). This retrospective study examined patients with brain metastasis who received LINAC-FSRT treatment at Jiangsu Cancer Hospital between January 2020 and December 2022. The inclusion criteria consisted of (1): confirmed primary tumor pathology; (2) pre-treatment magnetic resonance imaging (MRI) and computed tomography (CT) scans; (3) absence of progression in the extracranial primary tumor or any related extracranial metastases within the last three months; (4) no prior brain radiation therapy; (5) provision of informed consent and availability for follow-up. Patients underwent follow-up every 2-3 months post-FSRT via in-person or telephone consultations, encompassing documentation of adverse reactions and monitoring of survival outcomes. This study focuses on the evaluation of intracranial progression-free survival (iPFS), while also considering secondary endpoints such as local control (LC), intracranial local failure (ILF), and overall survival (OS). iPFS is defined as the duration from the initiation of FSRT to the occurrence of BM progression or the most recent follow-up in the absence of progression. LC shows no evidence of tumor progression or new metastases in the treated areas. ILF indicates advancement in areas that have been previously treated during the follow-up period. LC and ILF evaluations for BM adhere to the Response Evaluation Criteria in Solid Tumors version 1.1 criteria (18). This research employed the Mini-Mental State Examination (MMSE) to evaluate neurocognitive functions in patients prior to and at 4 and 12 months following FSRT. A score below 3 in memory and attention-related assessments suggests possible declines in these domains. Non-cognitive toxicities were assessed using patient reports and objective measures. Headaches and dizziness were identified from patient statements, whereas vision decline was evaluated through both patient descriptions and professional assessments. Diagnoses of epilepsy and paralysis relied on electroencephalograms and comprehensive neurological examinations. RN was identified by MRI according to established criteria: increased T1 signals accompanied by surrounding edema, stability or regression of the lesion over a 4-month period without additional treatment, and lack of perfusion in enhanced MRI regions, with recurrence considered if lesions expanded within a minimum of 4 months (19).

### 2.2 Design of the FSRT protocol

The FSRT protocols utilized in this study were developed in accordance with established clinical guidelines and consensus, integrating the clinical expertise of radiation therapists and specific patient conditions. The protocols included: 8 Gy × 3 Fx (71 patients), 6 Gy × 5 Fx (19 patients). 12 Gy × 2 Fx (8 patients), 6 Gy × 4 Fx (17 patients), 4 Gy × 10 Fx (40 patients), 5 Gy × 7 Fx (22 patients), 5 Gy × 5 Fx (5 patients), 6 Gy × 7 Fx (8 patients), 4 Gy × 8 Fx (5 patients), 8 Gy × 6 Fx (5 patients), 6 Gy × 8 Fx (8 patients), and 4 Gy × 12 Fx (9 patients) (17, 20). Patients were positioned in a supine manner, secured with a plastic mask, and underwent CT scans utilizing a 1 mm slice thickness for the purpose of tumor localization. MRI scans, with comparable slice thickness, were integrated with CT images to accurately delineate the gross tumor volume (GTV). The planning target volume (PTV) was defined by extending 0-2 mm from GTV (21). The treatment employed a 6 MV X-ray linear accelerator equipped with a 2.5 mm multileaf collimator (22). To enhance accuracy and efficacy, the criteria for treatment plans include (19, 22, 23): ≥ 95% PTV coverage by the prescribed dose; protecting organs at risk, particularly those with low tolerance such as the lens; a dose fall-off rate exceeding 10% per 3 mm; and the avoidance of overlap of 10 Gy isodose lines in adjacent target areas.

The HyTEC guidelines establish dose-volume constraints to maintain the risk of symptomatic RN in patients with BM below 10% (16, 17). They specifically restrict the volumes irradiated at 12 Gy (V12) to 5 cm³ for a single fraction, 20 Gy (V20) to 20 cm³ for 3 fractions, and 24 Gy (V24) to 20 cm³ for 5 fractions, based on the total brain volume, including PTV. This study evaluated the feasibility of appropriately reducing the HyTEC constraints by categorizing patients into standard and adjusted HyTEC protocol groups according to specific dose-volume tolerance criteria. Patients in the standard group adhered to the HyTEC guidelines, requiring one of the following conditions: V12 < 5 cm³ during 2-fraction treatment; V20 < 20 cm³ for 3-4 fractions; V24 < 20 cm³ for 5-8 fractions. The adjusted group, formed under reduced criteria, comprised patients who failed to meet the standard group requirements yet fulfilled one of the following conditions: V12−V_PTV_ < 5 cm³ in 2-fraction treatment; V20 − V_PTV_ < 20 cm³ in 3-4 fractions; V24 − V_PTV_ < 20 cm³ in 5-8 fractions.

This study evaluated dose-response associations with BED10, calculated via the linear-quadratic (LQ) model with an *α*/*β* ratio of 10, categorizing patients into groups receiving ≤ 55 Gy and > 55 Gy. Furthermore, we conducted a stratified subgroup analysis of patients receiving FSRT with TKIs to compare survival outcomes between these dosage groups. Additionally, the study assessed the impact of treatment timing by comparing concurrent (within a two-week interval) versus non-concurrent therapies.

### 2.3 Data analysis

Data were analyzed using SPSS version 26.0. Specifically, continuous variables were assessed using the Wilcoxon rank-sum test, whereas categorical variables were evaluated using the Chi-square test. Kaplan-Meier survival analysis and the log-rank test were employed to examine differences in iPFS and OS. To identify independent prognostic factors, both univariate and multivariate Cox proportional hazards regression models were applied. Statistical significance was defined as *P* < 0.05.

## 3 Results

### 3.1 Patient clinical and treatment characteristics

This research analyzed 214 individuals with brain metastases treated with LINAC-FSRT. The median Karnofsky Performance Status (KPS) score was 80 (range 60-100); the median age was 61 years (range 32–84); and males comprised 56.5% of the cohort. Non-small cell lung cancer (NSCLC) constituted the primary tumor in 79.0% of instances. In BM, the median lesion count was 1 (range 1–8); the maximum diameter was 1.605 cm (range 0.3–7.06 cm); and the volume was 3.16 cm³ (range 0.05–188.24 cm³). Treatments ranged from 24 Gy to 50 Gy delivered over 2 to 12 fractions, tailored by the treating physicians. **Table 1** summarizes the baseline characteristics of individuals stratified by different BED10 values and those adhering to diverse dose-volume limitations. In the BED10 categories, the median BED10 for all patients was 48 Gy (range 37.5–86.4 Gy). The standard HyTEC protocol cohort comprised 71 patients. The adjusted cohort included 45 patients, with median PTV volumes of 14.73 cm³ (range 2.92–76.03 cm³) for 3–4 fraction treatments and 14.84 cm³ (range 4.14–53.43 cm³) for 5–8 fraction treatments.

**Table 1:** Patient and tumor baseline characteristics.

Characteristic	BED10			Different Dose-Volume Constraint Criteria		
	≤ 55 Gy (N = 125)	> 55 Gy (N = 89)	*P*	Standard HyTEC Protocol Group (N = 71)	Adjusted HyTEC Protocol Group (N = 45)	*P*
Gender, NO. (%)			0.639			0.751
Female	56 (44.8)	37 (41.6)		31 (43.7)	21 (46.7)	
Male	69 (55.2)	52 (58.4)		40 (56.3)	24 (53.3)	
Age, years, median (range)	62 (32-84)	59 (32-81)	0.643	62 (32-84)	61 (44-74)	0.461
KPS, percentages, median (range)	80 (60-100)	80 (60-100)	0.822	90 (60-100)	90 (60-100)	0.396
Primary Site, NO. (%)			0.541			0.485
Esophagus	7 (5.6)	6 (6.7)		3 (4.2)	2 (4.4)	
Lung	98 (78.4)	71 (79.8)		59 (83.1)	35 (77.8)	
Breast	9 (7.2)	6 (6.7)		5 (7.0)	4 (8.9)	
Other	11 (8.8)	6 (6.7)		4 (5.6)	4 (8.9)	
Extracranial Disease Control, NO. (%)			0.325			0.341
Controlled	94 (75.2)	72 (80.9)		56 (78.9)	32 (71.1)	
Uncontrolled	31 (24.8)	17 (19.1)		15 (21.1)	13 (28.9)	
Number of BM, NO. (%)			**0.005**			**0.022**
1	68 (54.4)	66 (74.2)		52 (73.2)	24 (53.3)	
1-4	47 (37.6)	22 (24.7)		18 (25.4)	16 (35.6)	
> 4	10 (8.0)	1 (1.1)		1 (1.4)	5 (11.1)	
Maximum Diameter of BM, NO. (%)			0.153			
≤ 2 cm	80 (64.0)	51 (57.3)		66 (93.0)	22 (48.9)	
2-3 cm	27 (21.6)	22 (24.7)		5 (7.0)	16 (35.6)	
3-4 cm	13 (10.4)	6 (6.7)		0 (0.0)	6 (13.3)	
> 4 cm	5 (4.0)	10 (11.2)		0 (0.0)	1 (2.2)	
Volume of BM, NO. (%)			0.657			**0.001**
≤10 cm³	96 (76.8)	66 (74.2)		71 (100.0)	31 (68.9)	
> 10 cm³	29 (23.2)	23 (25.8)		0 (0.0)	14 (31.1)	
CNS Toxicity, NO. (%)			0.091			0.889
None	95 (76.0)	76 (85.4)		56 (78.9)	35 (77.8)	
Present	30 (24.0)	13 (14.6)		15 (21.1)	10 (22.2)	
Symptoms of CNS Before FSRT, NO. (%)			0.977			**0.008**
None	77 (61.6)	55 (61.8)		56 (78.9)	25 (55.6)	
Present	48 (38.4)	34 (38.2)		15 (21.1)	20 (44.4)	
TKIs Targeted Therapy, NO. (%)			0.86			0.885
None	80 (64.0)	58 (65.2)		48 (67.6)	31 (68.9)	
Received	45 (36.0)	31 (34.8)		23 (32.4)	14 (31.1)	
Immunotherapy, NO. (%)			0.285			0.916
None	82 (65.6)	52 (58.4)		48 (67.6)	30 (66.7)	
Received	43 (34.4)	37 (41.6)		23 (32.4)	15 (33.3)	

The bold values represent statistically significant differences with *P* < 0.05. BED10, biologically effective dose with an alpha/beta ratio of 10. N, number. KPS, karnofsky performance status. BM, brain metastases. CNS, central nervous system. TKIs, tyrosine kinase inhibitors. HyTEC, Hypofractionated Treatment Effects in the Clinic.

### 3.2 Survival and intracranial control outcomes

As of December 2023, the median follow-up for this cohort was 16.8 months (range 1.47–52.5 months). In this timeframe, 165 patients experienced disease progression, with 154 demonstrating intracranial advancement and an ILF rate of 23.4% (50/214). The LC rates following LINAC-FSRT at 3 and 12 months were 87.4% and 68.7%, respectively. The median iPFS was 12.4 months, with cumulative rates of 86% at 3 months and 50.5% at 12 months. Multivariate Cox analysis of iPFS (**Table 2**) revealed a substantial association between extracranial disease control and iPFS. Ninety-nine patients (46.3%) succumbed; the median OS was not attained, anticipated to surpass 16.4 months, with cumulative OS rates of 92.5% at 3 months and 69.6% at 12 months. Multivariate regression analysis (**Table 3**) revealed that increased age, uncontrolled extracranial illness, and greater BM volume significantly reduced OS, whereas higher BED10 doses considerably enhanced OS.

**Table 2:** Univariate and multivariate regression for iPFS.

Variable	Univariate Regression		Multivariate Regression	
	HR (95%CI)	*P*	HR (95%CI)	*P*
Age, years	1.013 (0.995-1.031)	0.166		
Gender	1.174 (0.852-1.618)	0.328		
KPS (> 70 vs ≤ 70)	0.865 (0.550-1.358)	0.528		
BED10, Gy (≤ 55 vs > 55)	0.823 (0.592-1.145)	0.248		
Number of BM, NO.		0.342		
2-4 vs 1	0.986 (0.703-1.383)	0.936		
≥ 4 vs 1	0.569 (0.249-1.300)	0.181		
Volume of BM, cm^3^ (> 10 vs ≤ 10)	1.405 (0.986-2.001)	0.060	1.418 (0.995-2.022)	0.054
Extracranial Disease Control (Yes vs No)	2.677 (1.872-3.829)	0.001	2.692 (1.880-3.853)	0.001
Symptoms of CNS Before FSRT (Yes vs No)	1.119 (0.811-1.545)	0.493		
Chemotherapy (Yes vs No)	1.034 (0.754-1.420)	0.835		
Immunotherapy (Yes vs No)	0.988 (0.713-1.369)	0.940		
TKIs Targeted Therapy (Yes vs No)	0.823 (0.592-1.145)	0.248		

In the univariate regression analysis for iPFS, factors with a p-value below 0.1 were included in the multivariate analysis (P < 0.05) to assess their adjusted effects on iPFS. Abbreviations: iPFS, intracranial progression-free survival. BED10, biologically effective dose with an alpha/beta ratio of 10. KPS, karnofsky performance status. BM, brain metastases. CNS, central nervous system. TKIs, tyrosine kinase inhibitors. FSRT, fractionated stereotactic radiotherapy. HR, hazard ratio. CI, confidence interval.

**Table 3:** Univariate and multivariate regression for OS.

Variable	Univariate Regression		Multivariate Regression	
	HR (95%CI)	*P*	HR (95%CI)	*P*
Age, years	1.030 (1.007-1.054)	0.011	1.030 (1.007-1.053)	0.010
Gender	1.314 (0.877-1.970)	0.186		
KPS (> 70 vs ≤ 70)	0.717 (0.419-1.225)	0.223		
BED10, Gy (≤ 55 vs > 55)	0.643 (0.422-0.978)	0.039	0.656 (0.431-0.998)	0.049
Number of BM, NO.		0.671		
2-4 vs 1	0.878 (0.571-1.350)	0.553		
≥ 4 vs 1	0.681 (0.248-1.872)	0.457		
Volume of BM, cm^3^ (> 10 vs ≤ 10)	1.770 (1.158-2.704)	0.008	1.789 (1.170-2.736)	0.007
Extracranial Disease Control (Yes vs No)	3.024 (1.993-4.589)	0.001	3.063 (1.987-4.722)	0.001
Symptoms of CNS Before FSRT (Yes vs No)	1.177 (0.787-1.759)	0.427		
Chemotherapy (Yes vs No)	0.774 (0.520-1.152)	0.207		
Immunotherapy (Yes vs No)	0.661 (0.432-1.012)	0.057		
TKIs Targeted Therapy (Yes vs No)	0.648 (0.421-0.996)	0.048	0.857 (0.549-1.338)	0.498

In the univariate regression analysis for OS, factors with a p-value below 0.05 were included in the multivariate analysis (P < 0.05) to assess their adjusted effects on OS. Abbreviations: OS, overall survival. BED10, biologically effective dose with an alpha/beta ratio of 10. KPS, karnofsky performance status. BM, brain metastases. CNS, central nervous system. TKIs, tyrosine kinase inhibitors. FSRT, fractionated stereotactic radiotherapy. HR, hazard ratio. CI, confidence interval.

**Figure 1:**
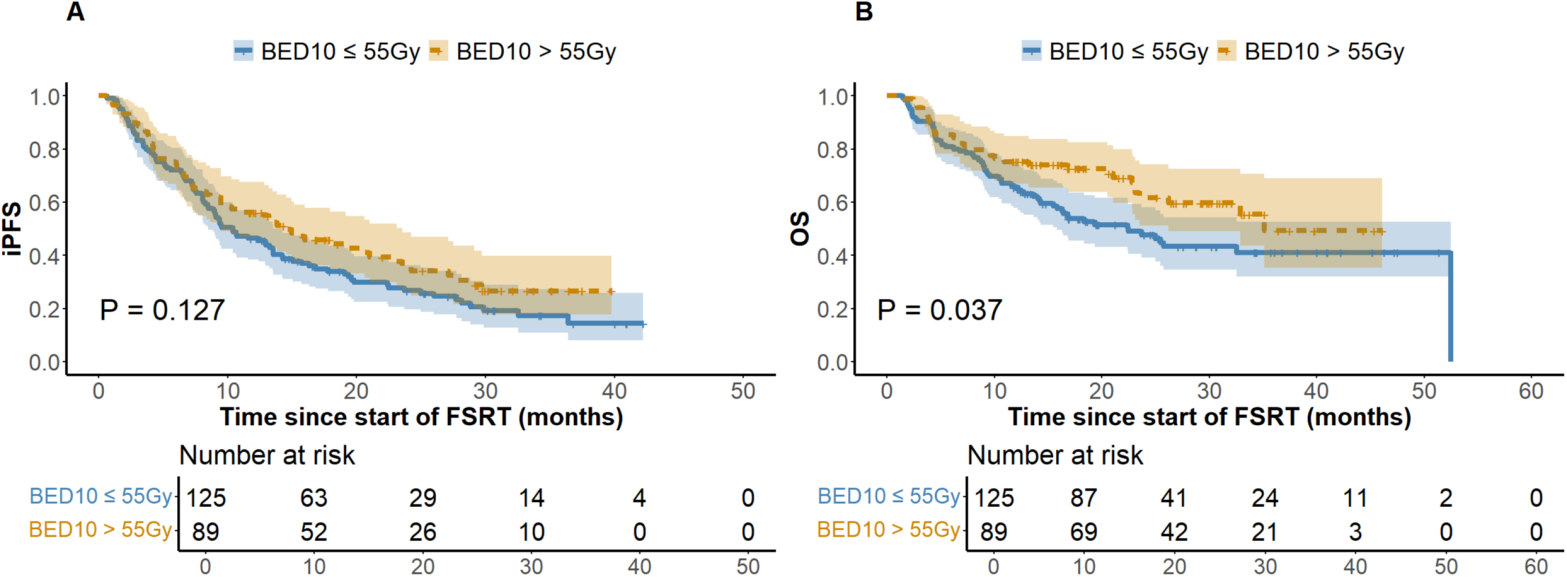
Kaplan-Meier Analysis of iPFS and OS by BED10 Dose Groups. **(A)** Intracranial Progression-free Survival. **(B)** Overall Survival. Abbreviations: iPFS, intracranial progression-free survival. OS, overall survival. BED10, biologically effective dose with an alpha/beta ratio of 10. FSRT, fractionated stereotactic radiotherapy.

**Figure 2:**
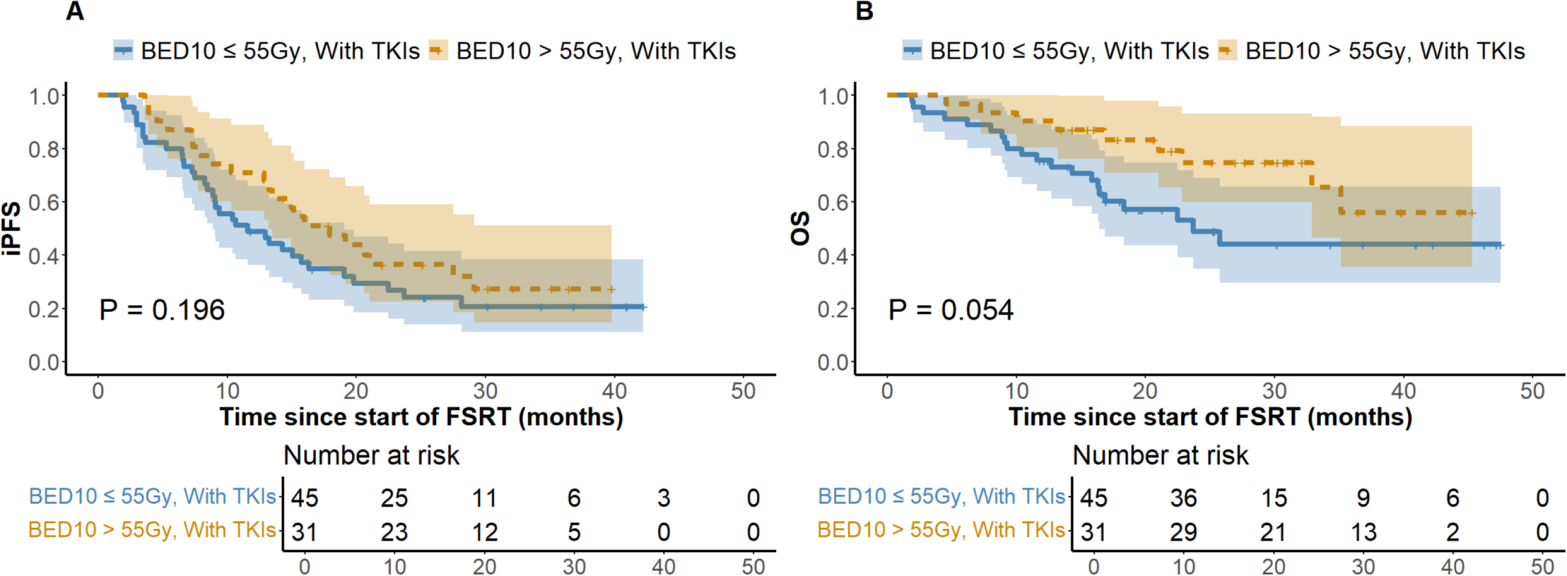
Kaplan-Meier Analysis of iPFS and OS in FSRT with TKIs by BED10 Dose. **(A)** Intracranial Progression-free Survival. **(B)** Overall Survival. Abbreviations: iPFS, intracranial progression-free survival. OS, overall survival. BED10, biologically effective dose with an alpha/beta ratio of 10. FSRT, fractionated stereotactic radiotherapy. TKIs, tyrosine kinase inhibitors. FSRT.

**Figure 3:**
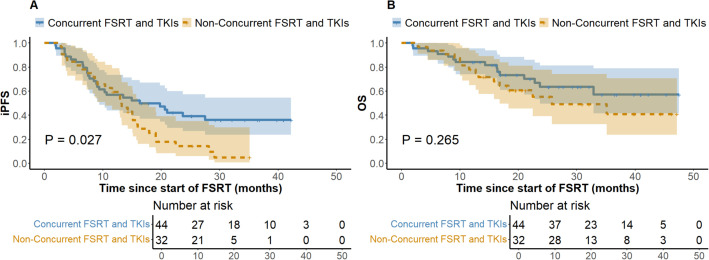
Kaplan-Meier Analysis of iPFS and OS by Therapy Timing in TKI-Treated Patients. **(A)** Intracranial Progression-free Survival. **(B)** Overall Survival. iPFS, intracranial progression-free survival. OS, overall survival. FSRT, fractionated stereotactic radiotherapy. TKIs, tyrosine kinase inhibitors. FSRT.

At the last follow-up, the ILF rates for the low (N = 125) and high (N = 89) BED10 groups were 24.8% and 22.5%, respectively (*P* = 0.694), while the one-year LC rates were 64.8% and 74.2% (*P =* 0.146). **Figure 1** depicts a one-year cumulative iPFS of 46.4% for BED10 ≤ 55 Gy and 56.2% for BED10 > 55 Gy, alongside one-year cumulative OS rates of 65.6% and 75.3%. No notable variation in iPFS was observed (*P =* 0.127), but OS exhibited a substantial enhancement in the high BED10 cohort (*P =* 0.037). In the non-TKIs subgroup (N = 138), no significant differences were observed in iPFS or OS between the low (N = 80) and high BED10 (N = 58) groups (*P =* 0.326 and *P =* 0.217), with one-year cumulative iPFS rates of 45.0% and 48.3%, and OS rates of 58.7% and 67.2%, respectively. In the FSRT coupled with TKIs subgroup (N = 76), no significant difference in iPFS was observed between BED10 ≤ 55 Gy (N = 45) and > 55 Gy (N = 31) (*P =* 0.196, **Figure 2A**), with one-year iPFS rates of 48.9% and 71.0%, respectively. OS differences neared significance (*P =* 0.054, **Figure 2B**), with one-year rates of 75.6% and 79.1%. The concurrent administration of TKIs markedly improved iPFS (*P =* 0.027), yielding one-year rates of 56.8% and 59.4%, however, OS exhibited no significant change (*P =* 0.265), with rates of 84.1% and 78.1%, respectively (**Figure 3**). This study analyzed survival outcomes in the non-lung cancer subgroup (45 patients), comparing those getting BED10 ≤ 55 Gy (27 patients) with those receiving BED10 > 55 Gy (18 patients), as illustrated in **Supplementary Figure 1**.

This study compared the prognosis of groups following different dose-volume constraints. The standard HyTEC protocol group exhibited a 16.9% ILF rate and a 66.2% one-year LC rate, while the adjusted group demonstrated rates of 22.2% and 64.8%, respectively. Chi-square analysis showed no significant differences in ILF and one-year LC rates. Survival analysis indicated one-year iPFS rates of 49.3% and 46.7% (*P =* 0.691), and OS rates of 64.8% and 75.9% for the standard and adjusted groups, respectively (*P =* 0.652). Additionally, patients were categorized by lesion volume into two groups: ≤ 4 cm³ (117 cases) and > 4 cm³ (97 cases); results are detailed in **Supplementary Table 1** and **Supplementary Figure 2**.

### 3.3 Central nervous system toxicity reactions

In a cohort of 214 patients undergoing LINAC-FSRT, 82 (38.3%) exhibited significant central nervous system (CNS) symptoms before treatment, with 74.4% achieving alleviation subsequently. Adverse events comprised dizziness and headaches in 21 patients (which resolved with mannitol), vision impairment in 12, memory decline in 13, cognitive dysfunction in 3, concentration challenges in 8, epilepsy in 2, hemiplegia in 4, and RN in 2. As per the Radiation Therapy Oncology Group (RTOG) CNS toxicity criteria, 17.3% experienced mild to moderate toxicity (grades 1-2), while 2.8% reported severe reactions (grades 3-4). Chi-square tests showed that high BED10, regardless of TKIs driver gene alterations, did not increase the likelihood of adverse effect (*P =* 0.180, *P =* 0.290). The administration of concurrent therapy did not increase the risk of post-FSRT CNS reactions (*P =* 0.339). Modifying the HyTEC protocol in the adjusted group did not substantially increase the chances of CNS toxicity (*P =* 0.889).

## 4 Discussion

In the medical field, BM substantially undermines patients’ quality of life and presents treatment obstacles due to its intricacy. FSRT, especially when utilizing LINAC, offers precise, high-dose radiation for multifocal, extensive, or architecturally intricate BM, while minimizing damage to surrounding healthy brain tissue. The availability of LINAC devices and associated insurance reimbursement have promoted the implementation of FSRT, meeting health economics considerations. This study evaluated the efficacy of LINAC-FSRT in treating BM, revealing a median iPFS of 12.4 months and an estimated median OS exceeding 16.4 months. These outcomes surpass previous reports, such as a study reporting a median OS of 13.2 months and iPFS of 6.3 months (24), as well as the JLGK0901 study, which reported median OS of 13.9 months for single BM and 10.8 months for 2-4 BM (25). Our investigation confirmed a 12-month LC rate of 68.7%, aligning with Thomsen et al. (26), who reported FSRT control rates between 65% (27) to 96% (28) for lesions over 2 cm. Consequently, our LINAC-FSRT demonstrates effective LC. Our findings underscore the potential of FSRT in managing BM, and comparison analyses between FSRT and SRS further validating its distinct benefits. For example, Fokas et al. discovered that FSRT and SRS achieved comparable median OS rates for larger metastases (*P* = 0.575), whereas SRS led to more adverse reactions (*P* = 0.01) (20). Minniti et al. reported similar OS for metastases larger than 2 cm, but FSRT had better LC (91% vs 77%, *P* = 0.01) and lower necrosis (9% vs 18%, *P* = 0.01) (4). According to Thomsen’s review, FSRT considerably reduces necrosis risk at comparable control levels (26). Remick et al. found no significant difference in LC rates or RN between SRS and FSRT. However, FSRT may improve LC when BED10 is ≥ 50 Gy (*P* = 0.09) (29). These studies illustrate FSRT’s promising function as an effective alternative to SRS in the treatment of BM. Further analysis identified uncontrolled extracranial disease as an independent risk factor for both iPFS and OS (**Tables 2**, **3**), implying that extracranial disease control may greatly influence BM survival, more so than factors like KPS, age, and volume (30, 31). Additionally, higher age and larger metastasis volumes correlated with poorer OS, consistent with recent prognostic assessments (30). Despite previous studies associating higher KPS with superior OS (25, 30, 32), in our cohort, where 85.2% had a KPS greater than 70, KPS did not impact OS, indicating the need for further classification. Different BM pathologies significantly affected prognosis (33), with primary melanoma patients typically having poorer outcomes. Notably, the majority (79.0%) with NSCLC responded better to treatment, reflecting advancements in patient selection and diagnosis at our facility.

While the efficacy and safety of FSRT for large-volume lesions are well established (34), many studies supporting its clinical value also encompass a considerable number of small-volume BM (20, 35). From a radiobiological perspective, the six Rs of fractionated radiotherapy facilitate FSRT in accurately regulating dose distribution, effectively target tumors, and minimize damage to adjacent normal brain tissue (36). We hypothesize that for small-volume lesions, FSRT conforming to HyTEC-recommended dose tolerance limits—by converting HyTEC’s single-fraction SRS dose limits to equivalent BED10—may demonstrate efficacy and safety comparable to SRS (17). To test this, we compared outcomes in patients with lesion volumes ≤ 4 cm³ to those with lesion volumes > 4 cm³. The small-volume group demonstrated significantly better iPFS and OS than the large-volume group (iPFS: *P* = 0.027; OS: *P*= 0.026) (**Supplementary Figure 2**). Nonetheless, one-year LC rates (*P*= 0.112) and the incidence of grade 3–4 CNS toxicities (*P*= 0.549) did not differ significantly between groups (**Supplementary Table 1**). The data indicate that FSRT is viable for the treatment of small-volume lesions and that cumulative lesion volume substantially affects prognosis. While direct comparative studies between FSRT and SRS for small-volume lesions are limited, the existing literature supports the potential of FSRT in this context, aligning with our findings. For instance, Michael et al. reported that FSRT for BM smaller than 1 cm attained a high LC rate of 86.8%, with a radiation necrosis incidence of only 3.2% (37). Kawai et al. confirmed the efficacy of 5-fraction FSRT for treating metastases less than 2 cm (38). Similarly, Marcrom et al. found that lesions smaller than 3 cm exhibited higher LC rates compared to larger lesions (95% vs. 75%), and that lesion size was positively correlated with post-treatment toxicity (*P*= 0.04) (39). In conclusion, we assert that applying FSRT to small-volume BM is both rational and potentially advantageous. Subsequent research should further verify the efficacy and safety of FSRT across varying lesion volumes to enhance therapeutic alternatives.

Recent studies have linked higher BED10 with improved intracranial LC, highlighting its potential value (40). A higher BED generally correlates with better LC rates, suggesting lower local failure rates. Our findings confirm this, indicating that patients with BED10 ≤ 55 Gy had elevated ILF rates (24.8% vs 22.5%) and diminished one-year LC rates (64.8% vs 74.2%) compared to those with BED10 > 55 Gy. Additionally, the elevated BED10 cohort demonstrated enhanced iPFS, as depicted in **Figure 1**. Studies utilizing tumor control models reveal that increasing BED enhances LC, with each additional 10 Gy reducing the hazard ratios (HR = 0.77, *P* = 0.009) (41). This conclusion is corroborated by Redmond et al. (42) and Dupic et al. (43). The LQ model i demonstrates a clear proportional relationship between dosage and tumor cell lethality. However, regions of high dosage exhibit considerable secondary effects, complicating dose-response relationships and possibly explaining the lack of significant LC differences observed among varying BED10 levels in our study. Consistent with Gu et al. (44), who found significant OS correlation at slightly lower BED levels (BED10 ≥ 50 Gy), our results identified BED10 > 55 Gy as an independent favorable predictor of OS. Despite suggestions that higher BED might increase CNS toxicity (42), our investigation revealed no substantial discrepancies in CNS adverse effects between the low and high BED groups. Given the predominance of NSCLC BM in our study, we conducted a thorough analysis of non-lung cancer patients. This subgroup analysis showed that patients receiving FSRT with BED10 > 55 Gy exhibited significantly improved OS compared to those receiving lower doses (*P* = 0.038) (refer to **Supplementary Figure 1**). High-dose FSRT appears to offer survival advantages across various histological variants of BM. While studies contrasting high and low dose of FSRT in non-lung primary are limited, previous studies on breast cancer and melanoma BM indirectly support this observation. These studies demonstrated clinical efficacy with FSRT regimens where BED10 values exceeded those employed in SRS or WBRT in corresponding trials (5, 45). In summary, our center’s experience with LINAC-FSRT supports that delivering higher radiation doses to tumor regions significantly enhances OS in BM patients while maintaining intracranial LC stability and safety. This may be attributed to the cumulative dose and repair differences between tumor and normal tissues across fractionations.

In the treatment of BM, TKI-targeted therapy is pivotal for enhancing outcomes and prolonging survival. Research indicates that patients with TKI-related driver mutations exhibit increased vulnerability to BM (9, 10), and those treated with TKIs frequently achieve substantial efficacy with limited harm to normal tissues, generally leading to improved prognoses (11). Theoretically, the combination of SRS with TKIs results in a transient breach of the blood-brain barrier, hence elevating medication concentrations in the cerebrospinal fluid. Additionally, TKIs alter tumor cell cycle distribution by increasing the proportion of cells in the G_2_/M and G_0_/G_1_ phases and enhancing radiation-induced damage, thereby enhancing radiotherapy effectiveness (12, 13). Notwithstanding restricted sample sizes, our stratified subgroup analysis of patients receiving TKI-targeted therapy revealed that the OS difference between the BED10 ≤ 55 Gy and > 55 Gy groups approached statistical significance (*P* = 0.054) (**Figure 2**), implying that high-dose radiotherapy may effectively enhance OS. Notably, the high BED10 group demonstrated a superior one-year iPFS rate (71.0% vs 48.9%), while among patients not receiving TKIs therapy, differences in one-year iPFS were minimal (45.0% vs 48.3%), indicating that iPFS may improve under high BED10 FSRT when combined with TKI-targeted therapy. Recent investigations confirm these synergistic effects. Deng et al. showed that SRS combined with TKIs improved OS compared to SRS alone (HR = 0.59, 95%CI: 0.49-0.71, *P* < 0.01) (46). Jia et al. observed enhanced OS with the combination of TKIs and SRS compared to WBRT in NSCLC BM (*P* = 0.042) (47). However, evidence comparing standalone TKIs therapy to combined SRT and TKIs is scant. Regarding the impact of combining TKIs with radiotherapy on the risk of RN, a consensus is lacking. Some studies suggest that TKIs augment radiosensitivity, leading to heightened damage and necrosis in normal brain tissue (48), whereas others demonstrate no substantial increase in RN with SRT combined with TKIs (47). In our study, no RN incidents were observed in patients treated with both treatments, possibly due to insufficient sample size and a median follow-up of about 16.8 months, which may be insufficient to identify RN as a long-term toxicity. Consequently, the prolonged safety of integrating TKIs with FSRT requires further comprehensive investigation. Our findings indicate possible clinical advantages of high-dose FSRT combined with TKIs, supporting the need for future research to determine accurate dose-response relationships through comprehensive trials. Moreover, considering the uncertainty of TKIs’ effective duration within the brain and the intricate short- and long-term consequences of FSRT, our analysis on treatment timing revealed that concurrent TKI therapy markedly prolonged iPFS without increasing CNS adverse effects (**Figure 3**), underscoring its active role in BM management, especially in intracranial disease control. This corresponds with Magnuson et al., who noted poorer OS with extended intervals between SRT and TKIs (49). Neither treatment cohort achieved median OS, and no significant differences in OS were observed, likely due to insufficient follow-up. Some studies show no marked survival improvement with synchronous treatment (50, 51), thus rendering the optimal timing of combined therapies a subject of contention.

Our study validates that FSRT effectively mitigates symptoms and attains substantial LC rates. However, the incidence of Grade 1–2 RTOG toxicities in our study was 17.3%, which is elevated compared to previously documented rates (25). Other studies reported that radiotherapy-related adverse effects ranged from 5% to 25% following SRT (52), indicating that toxicity assessments may vary due to study design and evaluation methods. The elevated incidence of Grade 1–2 toxicities in our study may be attributed to subjective assessment techniques, particularly concerning memory decline, cognitive dysfunction, and concentration challenges. While the MMSE provides quantitative data on neurocognitive status, variables such as personal emotions, physical condition, and testing environment may affect outcomes, perhaps leading to an overestimation of toxicity. For severe Grade 3–4 toxicities (incidence of 2.8%) and RN (two instances), we implemented more stringent, objective evaluations based on MRI findings and comprehensive clinical assessments, yielding more reliable safety data for FSRT. In comparison to prior studies, the RTOG 9005 trial established an acceptable upper threshold of 30% for the occurrence of Grade 3–5 CNS toxicity (53). A Phase II trial found a 4.4% incidence of irreversible Grade ≥ 3 neurologic damage only associated with radiation in SRS patients (54). A meta-analysis of 24 SRT trials indicated RN rates of 7.3% for lesions measuring 2–3 cm and 6.5% for lesions beyond 3 cm following FSRT (55). Based on these statistics, our incidences of Grade 3–5 toxicities and radiation necrosis are within acceptable ranges, indicating the safety of our FSRT regimen. Notably, RN, a significant issue following brain irradiation, generally manifests between 3 to 18 months after SRT and may be postponed for as long as 3 years (56). With a median follow-up of 16.8 months, the incidence of RN in our trial was remarkably low (0.93%), indicating that this toxicity may persist at a tolerable level over time. However, distinguishing tumor progression from RN remains challenging (57). Systemic therapies may also increase RN risk (58). Thus, these factors contribute to the uncertainty in assessing the risk of RN in our study. Evidence indicates a robust link between the dosage and the volumes of irradiated brain tissue in FSRT and the likelihood of CNS damage, including RN (4, 15). According to ASTRO criteria for FSRT fractionation, 27 Gy in 3 fractions or 30 Gy in 5 fractions is recommended for brain metastases approximately 20 cm³ (about 3.3 cm in diameter), which poses a challenge to HyTEC standards (17, 59). Our clinical practice explored FSRT plans ranging from 24–50 Gy delivered over 2–12 fractions, highlighting the constraints of HyTEC recommendations. We reassessed brain tissue tolerance by accounting for total brain volume minus PTV, allowing for more flexible dose-volume constraints in higher fraction schemes. For instance, we applied the tolerance standards for 1 fraction to 2-fraction treatments and 3-fraction standards to 4-fraction treatments. Patients with 6-8 fractions were treated according to 5-fraction standards. Our study tested the feasibility of relaxing HyTEC constraints. We found that, despite higher pre-treatment CNS symptoms in the adjusted group, post-treatment adverse reactions were not significantly different from the standard group, indicating safer than expected outcomes under relaxed constraints (**Table 1**). Principal indicators such as LC, OS, and iPFS exhibited stability. The median PTV volumes for 3-4 and 5-6 fraction treatments were 14.73 cm³ and 14.84 cm³, respectively, indicating that it is feasible to extend V20 and V24 limits by about these volumes. These results align with Minniti’s findings of a 6% risk of RN associated with normal brain tissue volumes ranging from 22.8 to 30.2 cm³ in three-fraction FSRT (4). Another study confirming the safety of a mean V24.4 (minus PTV) of 33.434 cm³ in five-fraction FSRT, yielding therapeutic advantages (60). Despite the variability in definitions of Vx, they offer valuable references for clinical practice. The adjusted group’s baseline characteristics included larger numbers of BM, volumes, and diameters (**Table 1**), often comprising patients with more intricate or severe diseases. This indicates that in BM treatment, particularly for volumes over 10 cm³, diameters over 4 cm, or numbers exceeding four, adherence to HyTEC standards may not be necessary, suggesting more adaptable criteria. While most studies limit FSRT dose-toxicity analysis to 1, 3, and 5 fractions, our study extends this to higher fractions (≥ 6), highlighting the necessity for more investigation to standardize and evaluate Vx-CNS toxicity responses across various fractionation protocols.

This study, limited by its single-center, retrospective nature, may exhibit selection bias. Its patient cohort might not fully capture the diversity of BM pathology or primary tumor types. In particular, most patients receiving targeted therapy combined with FSRT were patients with NSCLC in this study. Although our results suggest potential clinical benefits from high-dose FSRT combined with TKIs, caution is advised when generalizing these findings to other histological types. Care should be taken in assessing grade 1–2 toxicity risks to avoid misattributing non-treatment-related symptoms to therapy side effects. Future research should focus on utilizing standardized toxicity assessment methods and advanced imaging techniques to enhance the objectivity and accuracy of toxicity data. Our discussions on optimizing dose-tolerance volumes are based on theoretical models and clinical experience, rather than data from prospective trials. Therefore, recommendations for dose and fractionation schemes should be considered cautiously and validated in future clinical trials. Additionally, with a median follow-up of only 16.8 months, our study offers preliminary evidence of FSRT’s efficacy. However, the short duration may constrain our understanding of its long-term outcomes. We plan to extend follow-up in future research to thoroughly evaluate the long-term effects of FSRT on patients with BM.

## 5 Conclusions

LINAC-FSRT is recognized as a safe and effective treatment approach for BM. A BED10 greater than 55 Gy notably enhances OS and potentially improves LC. Combining high BED10 FSRT with targeted therapy may amplify synergistic effects. Concurrent targeted therapy significantly improves iPFS. Based on the HyTEC guidelines, it is feasible to moderately diminish the constraints for brain tissue tolerance volumes.

## Data Availability

The raw data supporting the conclusions of this article will be made available by the authors, without undue reservation.
