# Supplementary material for: Analysis of linear accelerator-based fractionated stereotactic radiotherapy in brain metastases: efficacy, safety, and dose tolerances
Source: Front Oncol. 2024 Nov 21;14:1471004. doi: 10.3389/fonc.2024.1471004 (PMC11647529; doi:10.3389/fonc.2024.1471004)
Supplement: Supplementary file 1 [file DataSheet1.docx]

Supplementary Material

# Supplementary Figures and Tables

## Supplementary Figures

**
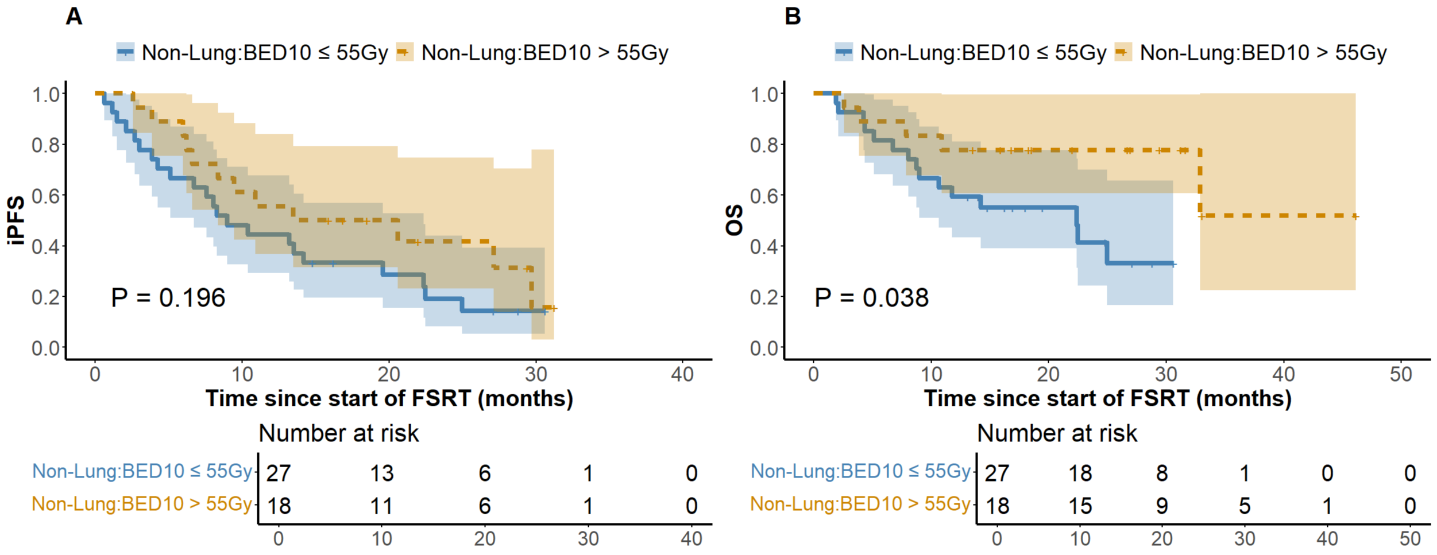
**

**Supplementary Figure 1.** **Survival Analysis Comparison between Non-Lung Cancer Patients with BED10 ≤ 55 Gy and BED10 > 55 Gy.** (A) Intracranial Progression-free Survival. (B) Overall Survival. Abbreviations: iPFS, intracranial progression-free survival. OS, overall survival. BED10, biologically effective dose with an alpha/beta ratio of 10. FSRT, fractionated stereotactic radiotherapy.

**
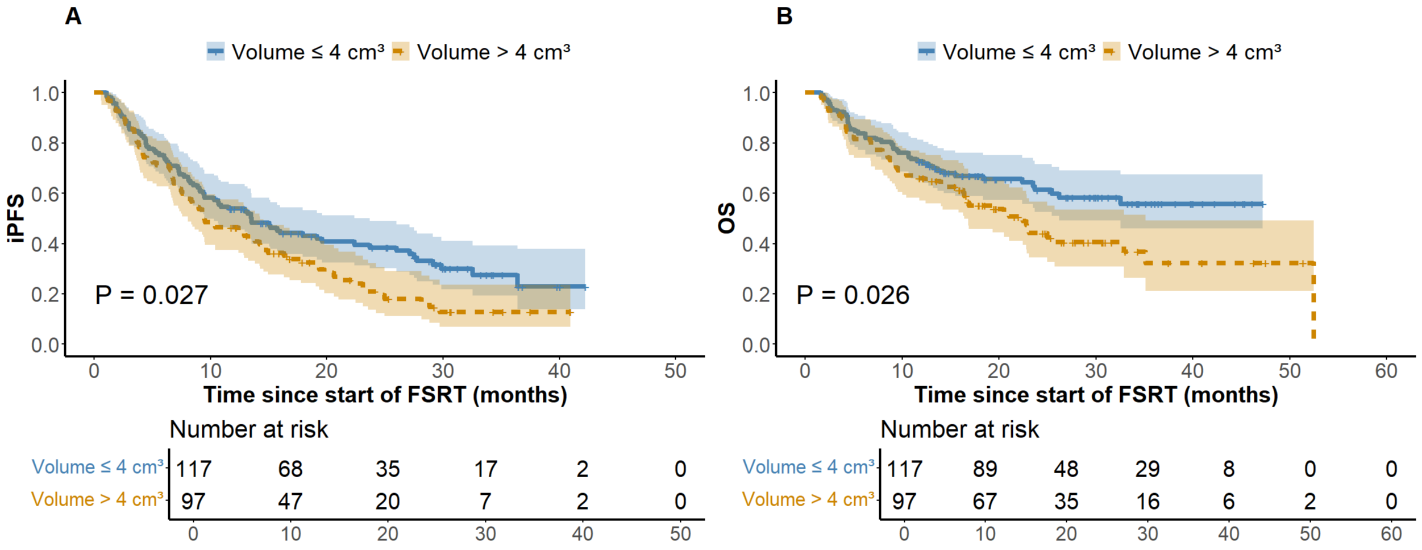
**

**Supplementary Figure 2.** **Survival Analysis of Patients by Tumor Volume: ≤4 cm³ vs >4 cm³.** (A) Intracranial Progression-free Survival. (B) Overall Survival. Abbreviations: iPFS, intracranial progression-free survival. OS, overall survival. BED10, biologically effective dose with an alpha/beta ratio of 10. FSRT, fractionated stereotactic radiotherapy.

## Supplementary Tables

**Supplementary Table 1: Comparison of Local Control and Toxicity in Patients with Tumor Volumes** $\leq$**4 cm³ and** $>$**4 cm³ Following FSRT.**

|  | Volume $\leq$ 4 cm³ Group (N=117) | Volume $>$ 4 cm³ Group (N=97) | *P* |
| --- | --- | --- | --- |
| one-year LC rates, % | 67.5 | 70.1 | 0.112 |
| ILF rates, % | 20.5 | 26.8 | 0.279 |
| Incidence of Grade 1-2 CNS Toxicity Reactions, % | 14.5 | 20.6 | 0.241 |
| Incidence of Grade 3-4 CNS Toxicity Reactions, % | 3.4 | 2.0 | 0.549 |

Abbreviations: LC, local control. CNS, central nervous system. ILF, Intracranial Local Failure. FSRT, fractionated stereotactic radiotherapy.
